# Synthesis, crystal structure and Hirshfeld surface analysis of di-μ_2_-chlorido-bis­[(nitrato-κ*O*)bis­(pyridine-κ*N*)copper(II)]

**DOI:** 10.1107/S2056989026010728

**Published:** 2026-10-30

**Authors:** Khadicha Toshboltaeva, Khayit Khudaynazarovich Turaev, Abdurasul Toshtemirov, Xolbek Zokirov

**Affiliations:** a Termez State University, Barkamol avlod str. 43, Termez, 190100, Uzbekistan; b University of Business and Science, Dostlik str. 14, Namangan, 160108, Uzbekistan; Vienna University of Technology, Austria

**Keywords:** crystal, copper, pyridine, synthesis, Hirshfeld surface analysis, hydrogen bonding

## Abstract

The title compound, [Cu_2_Cl_2_(NO_3_)_2_(C_5_H_5_N)_4_], crystallizes as a centrosymmetric binuclear Cu^II^ complex in which the two central Cu^II^ atoms are inter­connected by a pair of μ_2_-chlorido bridges. The Cu^II^ atom is additionally coordinated by two pyridine N atoms and one O atom from a monodentate nitrato ligand, resulting in a distorted square-pyramidal coordination environment. In the crystal, the binuclear complexes are linked by C—H⋯Cl and C—H⋯O hydrogen bonds, generating a supra­molecular framework structure. Hirshfeld surface analysis was performed to qu­antify and further elucidate the inter­molecular inter­actions.

## Abstract 2

The title compound is a centrosymmetric binuclear Cu^II^ complex, with one Cu^II^ ion, one chloride ligand, one nitrate ligand, and two pyridine ligands in the asymmetric unit.

## 1. Chemical context

Copper(II) complexes continue to attract considerable inter­est in coordination chemistry owing to the structural diversity of the Cu^II^ coordination environments and the ability of ancillary ligands to generate mono- and multinuclear structures. Pyridine is a simple neutral N-donor ligand that readily coordinates to Cu^II^ and has been widely employed in the preparation of many different copper(II) complexes (Pandey, 2018 ▸; Bowmaker *et al.*, 2011 ▸). In copper(II) halide–pyridine systems, the nature of the halido ligand(s) and the coordination environment can lead to distinct mol­ecular and extended structural motifs, in particular the ability of chlorido ligands to link symmetry-related copper(II) atoms through μ_2_-binding modes into binuclear units (Bowmaker *et al.*, 2011 ▸; Herringer *et al.*, 2011 ▸).
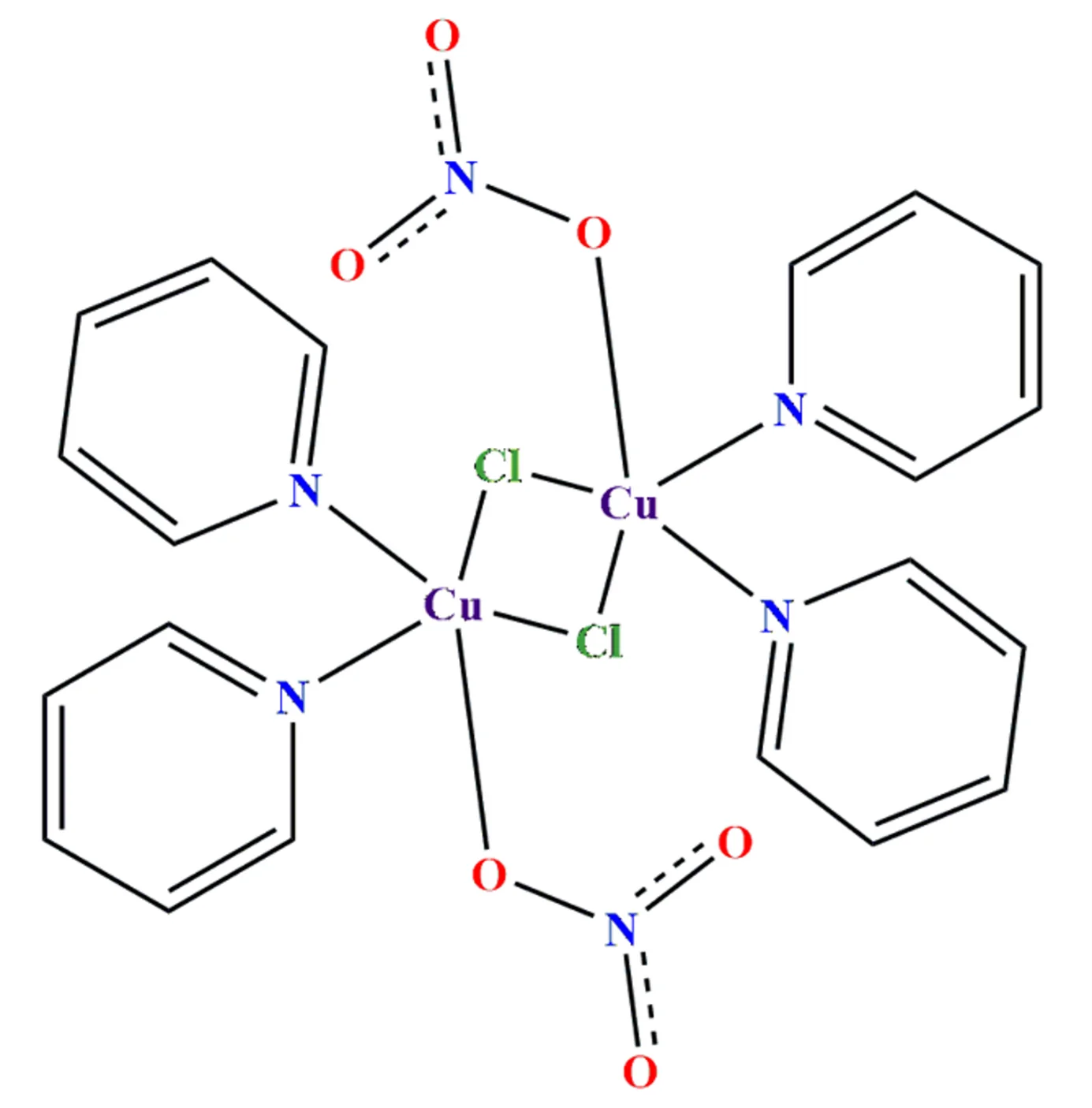


In this context, the present work describes the synthesis and single-crystal X-ray characterization of a centrosymmetric binuclear Cu^II^ complex, [Cu_2_(μ_2_-Cl)_2_(κO-NO_2_)_2_(C_5_H_5_N)_4_], (**1**), together with an analysis of its inter­molecular inter­actions.

## 2. Structural commentary

Compound (**1**) represents a centrosymmetric binuclear Cu^II^ mol­ecular complex in which two copper(II) ions are connected by two μ_2_-chlorido bridges, forming a {Cu_2_Cl_2_} core. The Cu^II^ atom is five-coordinate, with two ligating pyridine nitro­gen atoms (N1 and N2), one oxygen atom (O1) from a monodentate nitrate ligand, and the two bridging chloride ions (Fig. 1 ▸). The coordination environment of the Cu^II^ atom is distorted square-pyramidal, with the symmetry-related chlorido ligand Cl1(−*x* + 1, −*y* + 1, −*z* + 1) occupying the apical position. The five-coordinate geometry was assessed using the τ_5_ parameter (Addison *et al.*, 1984 ▸). The calculated value of τ_5_ = 0.18 indicates a shape closer to square-pyramidal than trigonal-bipyramidal, consistent with the observed structural parameters.

The observed distortion around Cu^II^ in compound (**1**) may be discussed in the context of the Jahn–Teller effect characteristic of many Cu^II^ complexes (Hakimov *et al.*, 2025 ▸). The Cu—N bond lengths are 1.998 (3) and 2.000 (3) Å, while the Cu—O1 and basal Cu—Cl1 distances are 2.021 (2) and 2.3009 (10) Å, respectively. The apical Cu1—Cl1(−*x* + 1, −*y* + 1, −*z* + 1) bond is significantly longer at 2.7025 (10) Å, and the intra­dimer Cu⋯Cu separation is 3.4958 (7) Å. The angles in the basal plane, N—Cu—N = 167.24 (13)° and O—Cu—Cl = 177.77 (8)°, deviate from ideal values.

## 3. Supra­molecular features

The crystal packing is consolidated by weak inter­molecular C—H⋯O and C—H⋯Cl contacts, the numerical parameters of which are summarized in Table 1 ▸. The C—H⋯O inter­actions connect neighbouring mol­ecules into layers parallel to (101). These layers are further linked by C—H⋯Cl contacts, resulting in a supra­molecular framework structure (Fig. 2 ▸). In addition, π–π contacts between pyridine rings within the centrosymmetric dimer are present, with centroid-to-centroid distances of 4.201 (2) Å between *Cg*3 (ring N1/C1–C5) and *Cg*4(–*x* + 1, –*y* + 1, –*z* + 1) (ring N2/C6–C10), and a slippage of 1.457 Å.

## 4. Hirshfeld surface analysis

A Hirshfeld surface analysis was performed using *CrystalExplorer* (Spackman *et al.*, 2021 ▸) to qu­antify the inter­molecular contacts in the crystal structure. The Hirshfeld surface mapped over *d*_norm_ is shown in Fig. 3 ▸. The red regions on the surface indicate close inter­molecular contacts, particularly C—H⋯O and C—H⋯Cl inter­actions, which contribute to the cohesion of the crystal packing. The two-dimensional fingerprint plots provide further insight into the relative contributions of the inter­molecular contacts (Fig. 4 ▸). H⋯H contacts show the largest contribution to the Hirshfeld surface. The prominent H⋯O/O⋯H contacts are associated with C—H⋯O inter­actions and are characterized by sharp features in the corresponding fingerprint plot (McKinnon *et al.*, 2007 ▸), consistent with directional close contacts. Similarly, the characteristic features in the H⋯Cl/Cl⋯H plot reflect inter­molecular C—H⋯Cl contacts. The H⋯C/C⋯H and O⋯C/C⋯O contributions arise from additional close contacts involving the aromatic rings and oxygen atoms. Although these contacts are less prominent in the fingerprint analysis, they provide further insight into the inter­molecular environment and the packing arrangement.

## 5. Database survey

A search of the Cambridge Structural Database (CSD, Version 2025.3.1; Groom *et al.*, 2016 ▸) for the di-*μ*_2_-chlorido-bis­[bis­(pyridine-κN)(nitrato-κO)copper(II)] complex unit resulted in three hits, CSD refcodes COLZOT (Han *et al.*, 2008 ▸), VAVXUM (Atria *et al.*, 2004 ▸) and ZATQOA (Alvarez-Larena *et al.*, 1995 ▸). These structures and that of compound (**1**) comprise two central Cu^II^ atoms linked through two *μ*_2_-chlorido ligands, forming a characteristic {Cu_2_Cl_2_} core, while each copper(II) atom is in a five-coordinate environment involving oxygen and nitro­gen donor atoms from the nitrate unit and different pyridine derivatives, respectively. Although COLZOT shares the Cu_2_Cl_2_ core with the title compound, its coordination environment differs significantly. In COLZOT, each Cu^II^ center is six-coordinate, with two pyridine nitrogen donors, two bridging chloride ions, one oxygen donor from a coordinated water molecule, and one oxygen donor from a nitrate ligand. This results in a distorted octahedral geometry, whereas the title compound contains five-coordinate Cu^II^ centers with a distorted square-pyramidal geometry. The key difference is the additional coordinated water molecule in COLZOT.

## 6. Synthesis and crystallization

Pyridine (2 mmol, 158 mg) and CuCl_2_·2H_2_O (1 mmol, 170 mg) were placed in a glass vial, followed by the addition of distilled water (4 ml) and concentrated HNO_3_ (1 ml). The mixture was stirred at room temperature for several minutes until a clear dark-blue solution was obtained. The solution was transferred to a 25 ml Teflon-lined stainless-steel autoclave, heated at 398 K for 48 h, and then cooled to 298 K at a rate of 10 K h^−1^ (Liu *et al.*, 2018 ▸). The resulting solution was transferred into a glass vial and kept in the dark at room temperature for four weeks, during which dark-blue prism-shaped crystals of [Cu_2_Cl_2_(C_5_H_5_N)_4_(NO_3_)_2_] formed. The crystals were isolated by filtration and dried at room temperature; yield 204 mg (64%, based on Cu), melting point 491–499 K.

## 7. Refinement

Crystal data, data collection and structure refinement details are summarized in Table 2 ▸. H atoms attached to C were positioned geometrically and refined using a riding model with distance constraints of C—H = 0.93 Å and with *U*_iso_(H) = 1.2*U*_eq_(C).

## Supplementary Material

Crystal structure: contains datablock(s) I. DOI: 10.1107/S2056989026010728/wm5808sup1.cif

Structure factors: contains datablock(s) I. DOI: 10.1107/S2056989026010728/wm5808Isup3.hkl

CCDC reference: 2494595

Additional supporting information:  crystallographic information; 3D view; checkCIF report

## Figures and Tables

**Figure 1:**
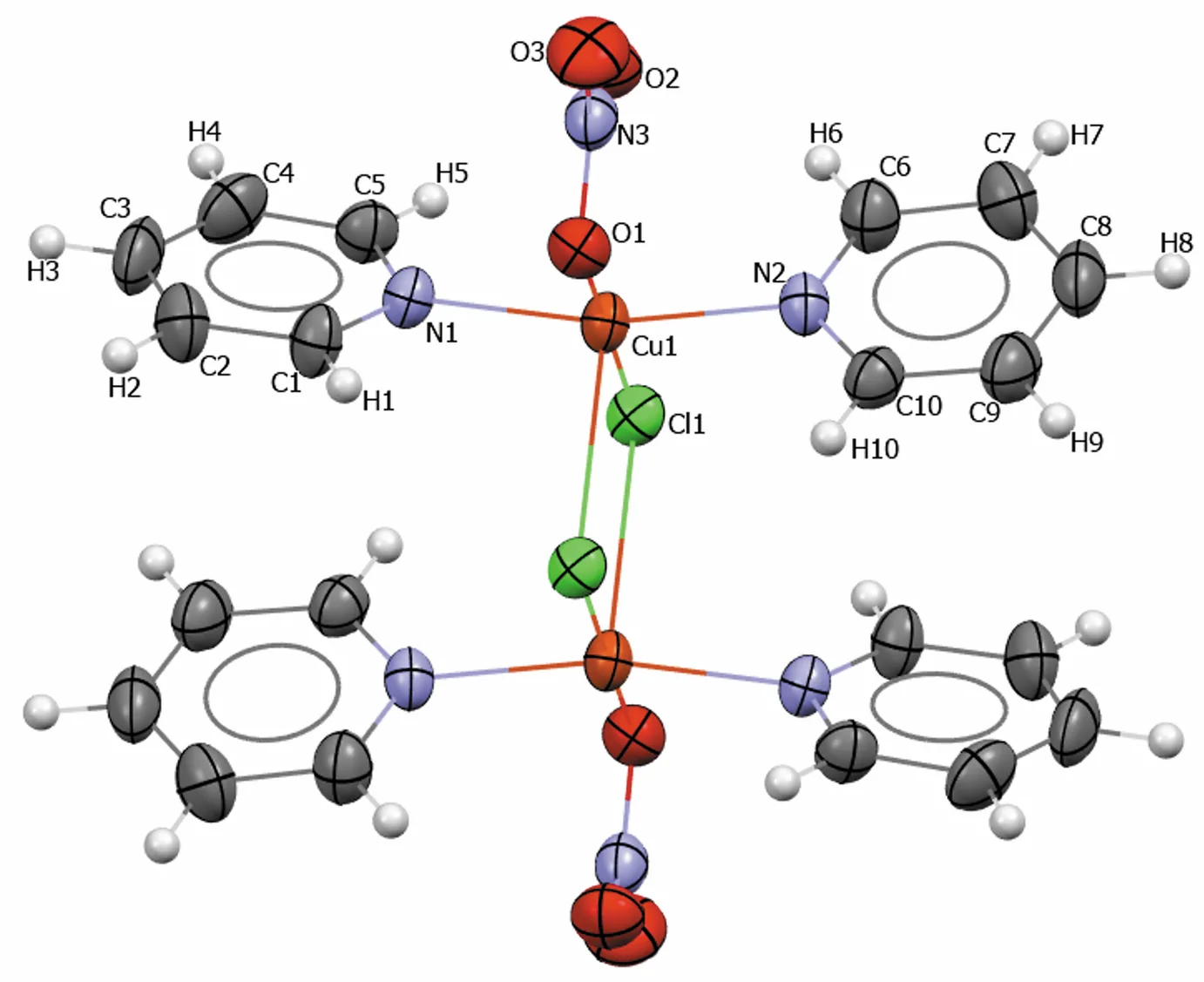
View of the title complex with the atom-numbering scheme of the asymmetric unit. Displacement ellipsoids are drawn at the 50% probability level; unlabelled atoms are related to the labelled atoms by symmetry operation −*x* + 1, −*y* + 1, −*z* + 1.

**Figure 2:**
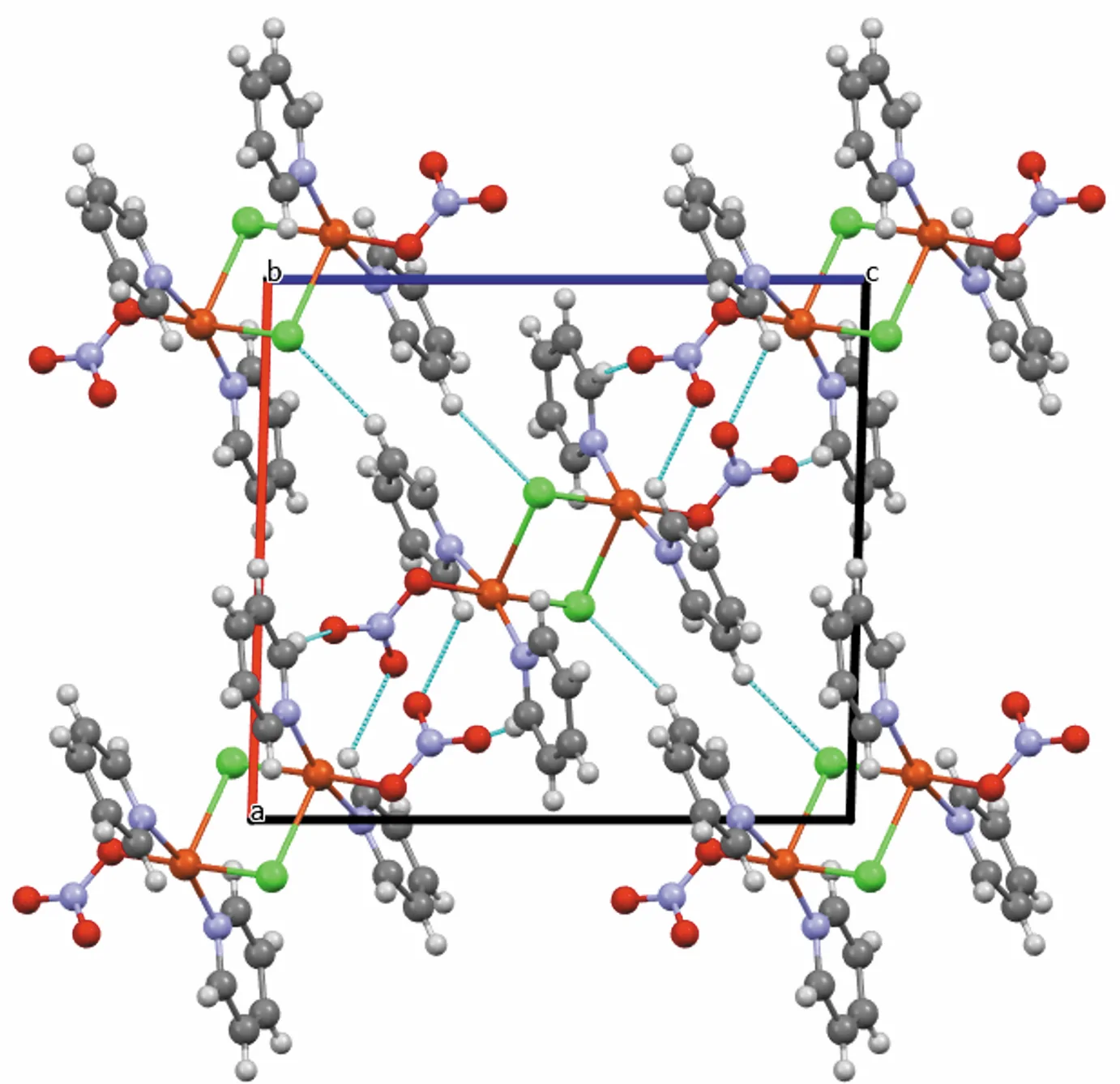
Packing plot of (**1**) in a view along [010]. C—H⋯O and C—H⋯Cl inter­actions are shown as turquoise dotted lines.

**Figure 3:**
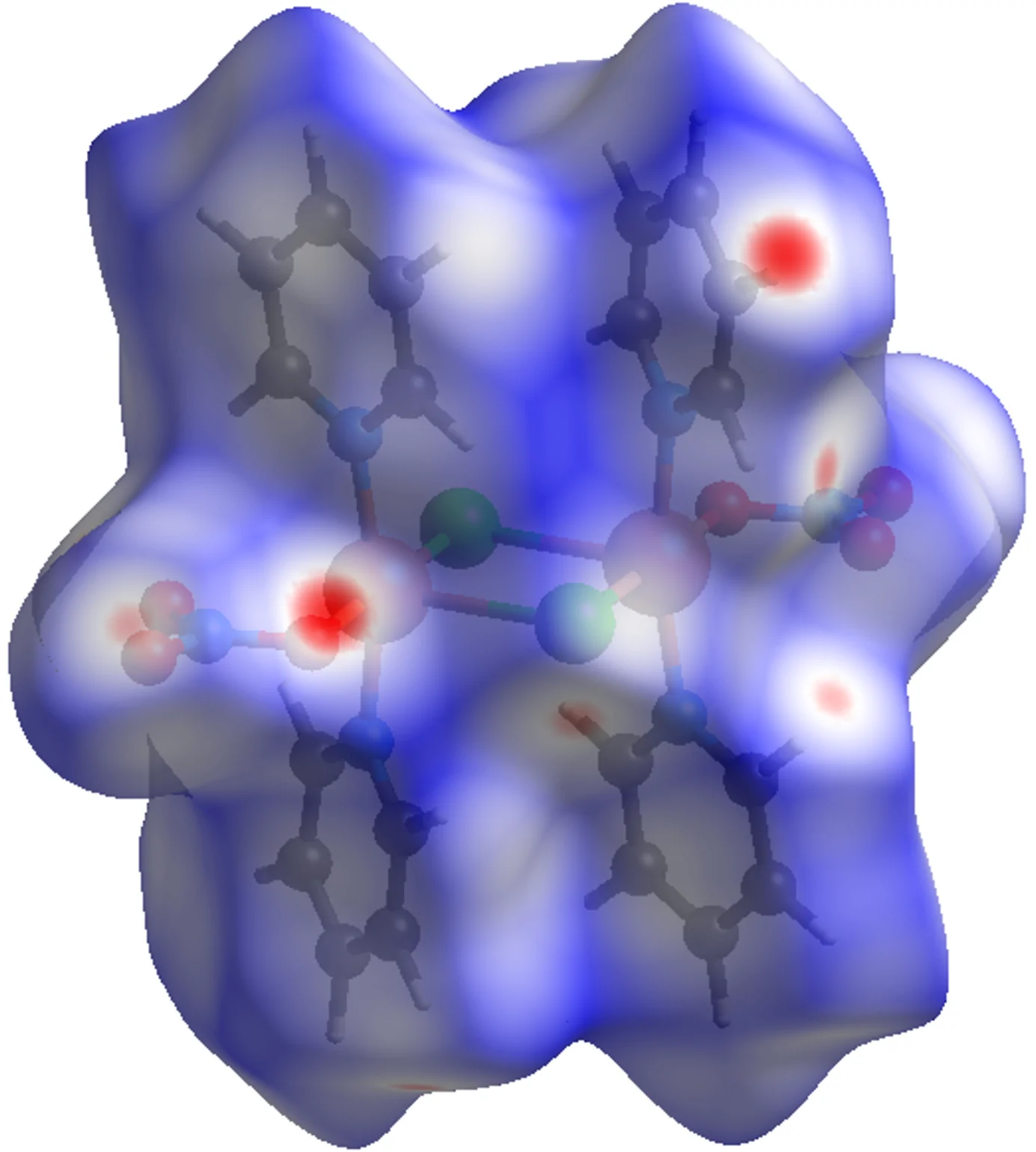
View of the three-dimensional Hirshfeld surface of the title compound plotted over *d*_norm_.

**Figure 4:**
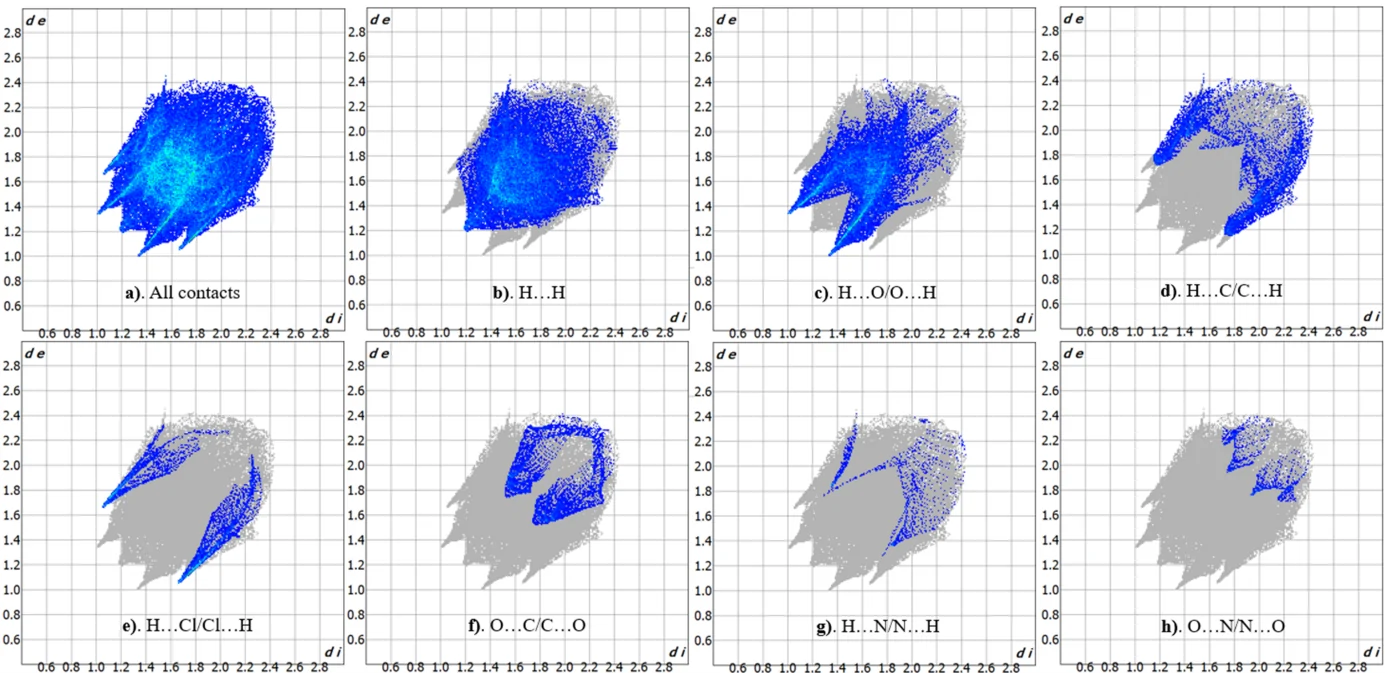
Two-dimensional fingerprint plots of (**1**) showing (*a*) all inter­actions, and delineated into (*b*) H⋯H (42.3%), (*c*) H⋯O/O⋯H (29.9%), (*d*) H⋯C/C⋯H (10.4%), (*e*) H⋯Cl/Cl⋯H (6.3%), (*f*) O⋯C/C⋯O (5.2%), (*g*) H⋯N/N⋯H (1.4%)and (*h*) O⋯N/N⋯O (1.4%) inter­actions.

**Table 1:** Hydrogen-bond geometry (Å, °)

*D*—H⋯*A*	*D*—H	H⋯*A*	*D*⋯*A*	*D*—H⋯*A*
C4—H4⋯O1^i^	0.93	2.49	3.390 (5)	163
C5—H5⋯O2^ii^	0.93	2.57	3.237 (4)	129
C2—H2⋯Cl1^iii^	0.93	2.84	3.639 (4)	135

Symmetry codes: (i) 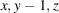; (ii) 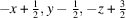; (iii) 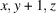.

**Table 2:** Experimental details

Crystal data	
Chemical formula	[Cu_2_Cl_2_(NO_3_)_2_(C_5_H_5_N)_4_]
*M*_r_	638.40
Crystal system, space group	Monoclinic, *P*2_1_/*n*
Temperature (K)	295
*a*, *b*, *c* (Å)	11.7560 (5), 8.1793 (3), 13.0074 (6)
β (°)	91.698 (4)
*V* (Å^3^)	1250.19 (9)
*Z*	2
Radiation type	Cu *K*α
μ (mm^−1^)	4.49
Crystal size (mm)	0.21 × 0.20 × 0.18

Data collection	
Diffractometer	Xcalibur, Ruby
Absorption correction	Multi-scan (*CrysAlis PRO*; Rigaku OD, 2020 ▸)
*T*_min_, *T*_max_	0.619, 1.000
No. of measured, independent and observed [*I* > 2σ(*I*)] reflections	3303, 1928, 1578
*R*_int_	0.026
θ_max_ (°)	62.0
(sin θ/λ)_max_ (Å^−1^)	0.573

Refinement	
*R*[*F*^2^ > 2σ(*F*^2^)], *wR*(*F*^2^), *S*	0.039, 0.103, 1.04
No. of reflections	1928
No. of parameters	164
H-atom treatment	H-atom parameters constrained
Δρ_max_, Δρ_min_ (e Å^−3^)	0.50, −0.34

Computer programs: *CrysAlis PRO* (Rigaku OD, 2020 ▸), *SHELXT* (Sheldrick, 2015*a* ▸), *SHELXL* (Sheldrick, 2015*b* ▸), *OLEX2* (Dolomanov *et al.*, 2009 ▸) and *publCIF* (Westrip, 2010 ▸).

## supplementary crystallographic information

### Di-µ2-chlorido-bis[(nitrato-κO)bis(pyridine-κN)copper(II)] Crystal data

**Table:**

[Cu_2_Cl_2_(NO_3_)_2_(C_5_H_5_N)_4_]	*F*(000) = 644
*M**_r_* = 638.40	*D*_x_ = 1.696 Mg m^−^^3^
Monoclinic, *P*2_1_/*n*	Cu *K*α radiation, λ = 1.54184 Å
*a* = 11.7560 (5) Å	Cell parameters from 1423 reflections
*b* = 8.1793 (3) Å	θ = 6.6–62.2°
*c* = 13.0074 (6) Å	µ = 4.49 mm^−^^1^
β = 91.698 (4)°	*T* = 295 K
*V* = 1250.19 (9) Å^3^	Block, clear blue
*Z* = 2	0.21 × 0.20 × 0.18 mm

### Di-µ2-chlorido-bis[(nitrato-κO)bis(pyridine-κN)copper(II)] Data collection

**Table:**

Xcalibur, Ruby diffractometer	1578 reflections with *I* > 2σ(*I*)
Detector resolution: 10.2576 pixels mm^-1^	*R*_int_ = 0.026
ω scans	θ_max_ = 62.0°, θ_min_ = 5.0°
Absorption correction: multi-scan (CrysAlisPro; Rigaku OD, 2020)	*h* = −10→13
*T*_min_ = 0.619, *T*_max_ = 1.000	*k* = −8→9
3303 measured reflections	*l* = −14→12
1928 independent reflections	

### Di-µ2-chlorido-bis[(nitrato-κO)bis(pyridine-κN)copper(II)] Refinement

**Table:**

Refinement on *F*^2^	Hydrogen site location: inferred from neighbouring sites
Least-squares matrix: full	H-atom parameters constrained
*R*[*F*^2^ > 2σ(*F*^2^)] = 0.039	*w* = 1/[σ^2^(*F*_o_^2^) + (0.0515*P*)^2^] where *P* = (*F*_o_^2^ + 2*F*_c_^2^)/3
*wR*(*F*^2^) = 0.103	(Δ/σ)_max_ = 0.001
*S* = 1.04	Δρ_max_ = 0.50 e Å^−^^3^
1928 reflections	Δρ_min_ = −0.34 e Å^−^^3^
164 parameters	Extinction correction: SHELXL2019/2 (Sheldrick, 2015b), Fc^*^=kFc[1+0.001xFc^2^λ^3^/sin(2θ)]^-1/4^
0 restraints	Extinction coefficient: 0.0040 (3)
Primary atom site location: iterative	

### Di-µ2-chlorido-bis[(nitrato-κO)bis(pyridine-κN)copper(II)] Special details

**Table:**

Geometry. All esds (except the esd in the dihedral angle between two l.s. planes) are estimated using the full covariance matrix. The cell esds are taken into account individually in the estimation of esds in distances, angles and torsion angles; correlations between esds in cell parameters are only used when they are defined by crystal symmetry. An approximate (isotropic) treatment of cell esds is used for estimating esds involving l.s. planes.

### Di-µ2-chlorido-bis[(nitrato-κO)bis(pyridine-κN)copper(II)] Fractional atomic coordinates and isotropic or equivalent isotropic displacement parameters (Å^2^)

**Table:**

	*x*	*y*	*z*	*U*_iso_*/*U*_eq_	
Cu1	0.41702 (4)	0.50764 (6)	0.60920 (4)	0.0344 (2)	
Cl1	0.39492 (7)	0.34861 (10)	0.46408 (6)	0.0378 (3)	
O1	0.4407 (2)	0.6529 (3)	0.73372 (19)	0.0417 (6)	
O2	0.2887 (2)	0.5160 (3)	0.7709 (2)	0.0531 (8)	
N1	0.5022 (2)	0.3299 (3)	0.6834 (2)	0.0362 (7)	
N2	0.3024 (3)	0.6668 (3)	0.5523 (2)	0.0373 (7)	
O3	0.3510 (3)	0.7100 (4)	0.8724 (2)	0.0644 (9)	
N3	0.3575 (3)	0.6260 (4)	0.7945 (2)	0.0426 (8)	
C1	0.6025 (3)	0.3566 (5)	0.7304 (3)	0.0441 (10)	
H1	0.633957	0.460729	0.727360	0.053*	
C10	0.3302 (4)	0.8169 (5)	0.5222 (3)	0.0445 (10)	
H10	0.405587	0.850344	0.529814	0.053*	
C5	0.4567 (3)	0.1802 (5)	0.6898 (3)	0.0450 (10)	
H5	0.386417	0.160124	0.657634	0.054*	
C6	0.1935 (4)	0.6214 (5)	0.5431 (3)	0.0519 (11)	
H6	0.172908	0.517588	0.564971	0.062*	
C9	0.2519 (4)	0.9237 (5)	0.4803 (3)	0.0559 (12)	
H9	0.274166	1.027395	0.459501	0.067*	
C4	0.5108 (4)	0.0548 (5)	0.7428 (4)	0.0591 (12)	
H4	0.477106	−0.047810	0.746832	0.071*	
C3	0.6148 (4)	0.0839 (5)	0.7891 (3)	0.0599 (13)	
H3	0.653637	0.000928	0.824276	0.072*	
C8	0.1408 (4)	0.8762 (6)	0.4695 (3)	0.0581 (12)	
H8	0.086473	0.946167	0.440424	0.070*	
C2	0.6609 (4)	0.2379 (6)	0.7828 (3)	0.0571 (12)	
H2	0.731328	0.260742	0.814085	0.069*	
C7	0.1111 (4)	0.7233 (6)	0.5025 (3)	0.0591 (12)	
H7	0.035716	0.688927	0.497299	0.071*	

### Di-µ2-chlorido-bis[(nitrato-κO)bis(pyridine-κN)copper(II)] Atomic displacement parameters (Å^2^)

**Table:**

	*U*^11^	*U*^22^	*U*^33^	*U*^12^	*U*^13^	*U*^23^
Cu1	0.0372 (4)	0.0333 (3)	0.0324 (4)	0.0025 (2)	−0.0061 (2)	0.0012 (2)
Cl1	0.0398 (5)	0.0382 (5)	0.0350 (5)	−0.0069 (4)	−0.0046 (4)	−0.0019 (4)
O1	0.0409 (15)	0.0408 (14)	0.0435 (15)	−0.0016 (12)	0.0011 (12)	−0.0033 (12)
O2	0.0487 (18)	0.0548 (17)	0.0559 (19)	−0.0097 (15)	0.0026 (14)	−0.0025 (14)
N1	0.0401 (18)	0.0338 (17)	0.0344 (17)	0.0018 (14)	−0.0053 (13)	0.0036 (13)
N2	0.0387 (18)	0.0362 (17)	0.0365 (17)	0.0071 (14)	−0.0064 (13)	0.0002 (14)
O3	0.085 (2)	0.0627 (19)	0.0459 (18)	0.0058 (17)	0.0107 (16)	−0.0184 (16)
N3	0.047 (2)	0.0409 (18)	0.0398 (19)	0.0111 (17)	−0.0020 (16)	0.0024 (16)
C1	0.049 (2)	0.038 (2)	0.045 (2)	−0.0009 (19)	−0.0158 (19)	0.0025 (18)
C10	0.051 (2)	0.038 (2)	0.045 (2)	0.0025 (19)	0.0019 (19)	0.0039 (19)
C5	0.043 (2)	0.042 (2)	0.051 (2)	−0.0078 (19)	−0.0021 (18)	0.0065 (19)
C6	0.051 (3)	0.044 (2)	0.061 (3)	0.002 (2)	−0.008 (2)	0.006 (2)
C9	0.068 (3)	0.042 (2)	0.058 (3)	0.014 (2)	0.006 (2)	0.010 (2)
C4	0.077 (3)	0.041 (2)	0.060 (3)	−0.004 (2)	0.004 (2)	0.013 (2)
C3	0.076 (3)	0.048 (3)	0.055 (3)	0.025 (2)	−0.003 (2)	0.017 (2)
C8	0.064 (3)	0.061 (3)	0.049 (3)	0.027 (3)	−0.006 (2)	0.005 (2)
C2	0.051 (3)	0.063 (3)	0.056 (3)	0.010 (2)	−0.016 (2)	0.006 (2)
C7	0.045 (3)	0.066 (3)	0.066 (3)	0.006 (2)	−0.012 (2)	0.000 (2)

### Di-µ2-chlorido-bis[(nitrato-κO)bis(pyridine-κN)copper(II)] Geometric parameters (Å, º)

**Table:**

Cu1—Cl1^i^	2.7025 (10)	C10—C9	1.370 (5)
Cu1—Cl1	2.3009 (10)	C5—H5	0.9300
Cu1—O1	2.021 (2)	C5—C4	1.380 (6)
Cu1—N1	1.998 (3)	C6—H6	0.9300
Cu1—N2	2.000 (3)	C6—C7	1.372 (5)
O1—N3	1.295 (4)	C9—H9	0.9300
O2—N3	1.242 (4)	C9—C8	1.366 (6)
N1—C1	1.329 (4)	C4—H4	0.9300
N1—C5	1.341 (4)	C4—C3	1.368 (6)
N2—C10	1.332 (5)	C3—H3	0.9300
N2—C6	1.335 (5)	C3—C2	1.375 (6)
O3—N3	1.228 (4)	C8—H8	0.9300
C1—H1	0.9300	C8—C7	1.371 (6)
C1—C2	1.361 (5)	C2—H2	0.9300
C10—H10	0.9300	C7—H7	0.9300
			
Cl1—Cu1—Cl1^i^	91.74 (3)	C9—C10—H10	118.8
O1—Cu1—Cl1^i^	86.11 (8)	N1—C5—H5	118.9
O1—Cu1—Cl1	177.77 (8)	N1—C5—C4	122.1 (4)
N1—Cu1—Cl1	91.59 (9)	C4—C5—H5	118.9
N1—Cu1—Cl1^i^	94.77 (9)	N2—C6—H6	119.0
N1—Cu1—O1	89.17 (11)	N2—C6—C7	122.0 (4)
N1—Cu1—N2	167.24 (13)	C7—C6—H6	119.0
N2—Cu1—Cl1	90.34 (9)	C10—C9—H9	120.4
N2—Cu1—Cl1^i^	97.79 (10)	C8—C9—C10	119.2 (4)
N2—Cu1—O1	89.38 (11)	C8—C9—H9	120.4
Cu1—Cl1—Cu1^i^	88.26 (3)	C5—C4—H4	120.6
N3—O1—Cu1	107.5 (2)	C3—C4—C5	118.9 (4)
C1—N1—Cu1	121.9 (2)	C3—C4—H4	120.6
C1—N1—C5	118.1 (3)	C4—C3—H3	120.6
C5—N1—Cu1	120.0 (2)	C4—C3—C2	118.8 (4)
C10—N2—Cu1	122.7 (3)	C2—C3—H3	120.6
C10—N2—C6	118.2 (3)	C9—C8—H8	120.7
C6—N2—Cu1	119.1 (3)	C9—C8—C7	118.7 (4)
O2—N3—O1	118.0 (3)	C7—C8—H8	120.7
O3—N3—O1	118.4 (3)	C1—C2—C3	119.3 (4)
O3—N3—O2	123.6 (4)	C1—C2—H2	120.4
N1—C1—H1	118.6	C3—C2—H2	120.4
N1—C1—C2	122.8 (4)	C6—C7—H7	120.3
C2—C1—H1	118.6	C8—C7—C6	119.4 (4)
N2—C10—H10	118.8	C8—C7—H7	120.3
N2—C10—C9	122.5 (4)		
			
Cu1—O1—N3—O2	5.0 (4)	N2—C6—C7—C8	0.4 (7)
Cu1—O1—N3—O3	−175.2 (2)	C1—N1—C5—C4	−0.4 (6)
Cu1—N1—C1—C2	178.8 (3)	C10—N2—C6—C7	1.1 (6)
Cu1—N1—C5—C4	−178.1 (3)	C10—C9—C8—C7	0.9 (7)
Cu1—N2—C10—C9	178.0 (3)	C5—N1—C1—C2	1.2 (6)
Cu1—N2—C6—C7	−178.5 (3)	C5—C4—C3—C2	1.1 (7)
N1—C1—C2—C3	−0.8 (7)	C6—N2—C10—C9	−1.5 (6)
N1—C5—C4—C3	−0.7 (7)	C9—C8—C7—C6	−1.4 (7)
N2—C10—C9—C8	0.5 (6)	C4—C3—C2—C1	−0.4 (7)

Symmetry code: (i) −*x*+1, −*y*+1, −*z*+1.

### Di-µ2-chlorido-bis[(nitrato-κO)bis(pyridine-κN)copper(II)] Hydrogen-bond geometry (Å, º)

**Table:**

*D*—H···*A*	*D*—H	H···*A*	*D*···*A*	*D*—H···*A*
C4—H4···O1^ii^	0.93	2.49	3.390 (5)	163
C5—H5···O2^iii^	0.93	2.57	3.237 (4)	129
C2—H2···Cl1^iv^	0.93	2.84	3.639 (4)	135

Symmetry codes: (ii) *x*, *y*−1, *z*; (iii) −*x*+1/2, *y*−1/2, −*z*+3/2; (iv) *x*, *y*+1, *z*.
